# Study on the characteristics of induced airflow and particle dispersion based on the multivariate two-factor model

**DOI:** 10.1371/journal.pone.0263740

**Published:** 2022-02-08

**Authors:** Chaonan Fan, Qingjie Qi, Xi Chen, Shaocheng Ge

**Affiliations:** 1 College of Safety Engineering and Technology, Liaoning Technical University, Fuxin Liaoning, China; 2 College of safety and emergency management engineering, Taiyuan University of Technology, Shanxi Taiyuan, China; Tianjin University, CHINA

## Abstract

To examine the diffusion characteristics of airflow and dust particles, a multi-factor and multi-level physical self-developed testing system is established. In this study, bunker height, chute angle, feeding speed, coal granularity, and belt speed are selected as independent variables, and airflow velocity and dust concentration are the response variables. The two-factor interactive model is established to analyze the primary and secondary relationship between the independent variables and the response variables. The results demonstrate a denser contour distribution of three-dimensional curved surfaces, suggesting an obvious interaction between the factors. The bunker height increases from 0.75 m to 1.15 m, the maximum increment of the induced airflow velocity at the outlet of the guide chute is observed to be 0.35 m/s, meanwhile, and with the increase in the feed speed from 2t/h to 8t/h, the increment of the induced airflow velocity at the outlet of the guide chute is recorded to be 51%. The coal granularity and bunker height depicted the highest influence on induced air velocity and dust concentration, and the feeding speed proved to be the secondary parameter. This two-factor interactive model can accurately forecast the actual values with a deviation of the calculated values limited to 9%. These research results support the existing research and provide a theoretical foundation to guide the dust control at belt conveyor transfer stations.

## 1. Introduction

**Fig 1:**
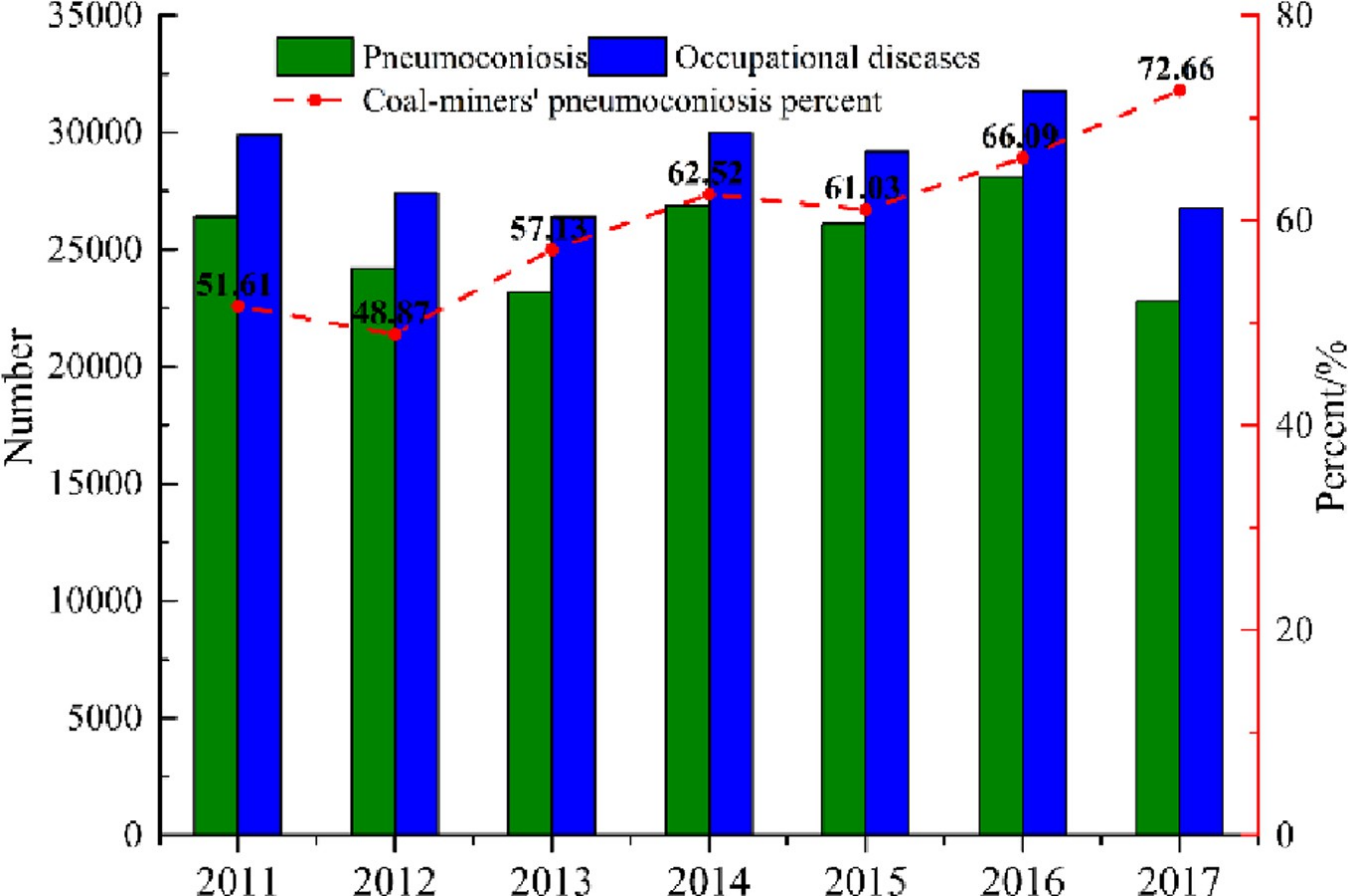
National occupational disease report of 2011~2017.

Conveyor transfer stations are extensively employed in the conveying processes, particularly those associated with mining and mineral processes. By the joint effort of induced airflow and shock wave, a large amount of dust particles is carried from the conveyor to other working areas, which is gradually becoming an alarming environmental problem. Such dust emissions not only cause great harm to the life and health of miners but also threaten the safety and efficiency of production [1–4]. Currently, China is one of the countries that is severely affected by dust in the world since its large population is affected by pneumoconiosis and dust-exposed diseases. Since the 1950s, there are 749,970 cases of occupational diseases, out of which, 676,541 cases of pneumoconiosis are recorded, and 149,110 death cases are found. Besides, 62% of its occupational pneumoconiosis victims are coal miners. According to the national occupational disease report of 2011 to 2017 issued by the National Health Commission of the People’s Republic of China, as highlighted in Fig 1, there are 26,756 cases of newly diagnosed occupational diseases in 2017, which includes 22,790 cases of occupational pneumoconiosis. These cases have exhibited an increase of 3721 cases from the last year. Moreover, the situation of pneumoconiosis in China is still critical, and new cases of pneumoconiosis are increasing quickly every year. Such a trend of the characteristics of occupational pneumoconiosis is centralized in the coal industries, occupations, and onset age [5–7].

**Fig 2:**
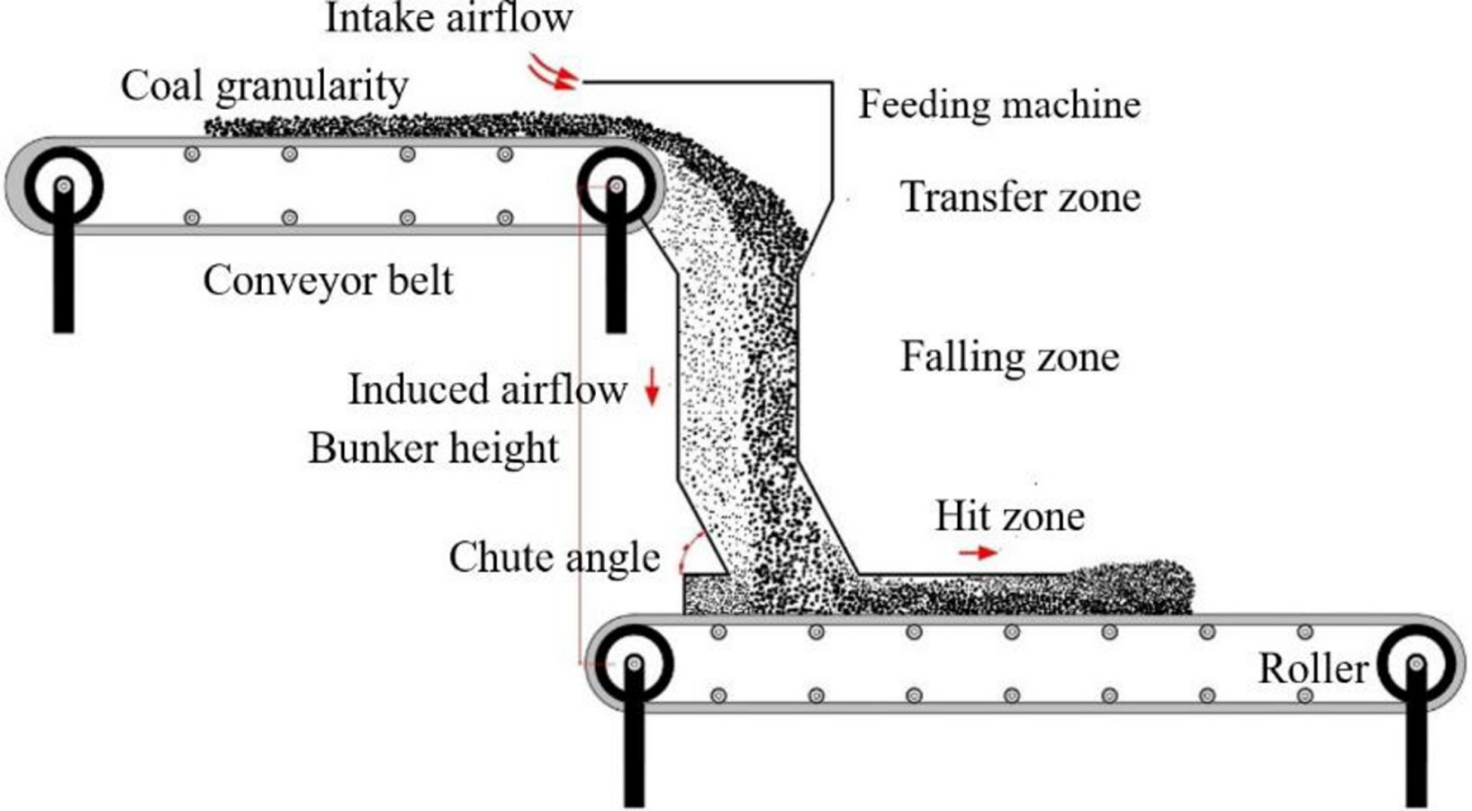
Dust generation processes of the falling stream.

The complexity of the process of induced airflow and dust diffusing at transshipment point varies hugely by various factors in different falling regions [8, 9]. There are three time zones in the process of coal transfer: transfer zone, falling zone, and hit zone. This is shown in Fig 2. Out of all the working zones involved in different periods, more than 80% of the respirable dust is produced due to the induced airflow, which is observed primarily in the following aspects: ① the induced airflow carries dust particles spread around in the blanking tube when the lump coal is dropped. ② When the induced airflow and lump coal fall together to hit the lower belt, the direction of motion of both are compelled to change, which stirs up the dust particles adhered to the belt surface. ③ The dust particles carried by induced airflow spit out from the outlet of skirt plate [10–12].

Very little research is done on the induced airflow and dust diffusion in the process of transferring. Hemeon is the first to obtain the relationship between the free-falling particle stream and the induced airflow. This relationship was validated by modeling a theoretical calculation formula of the induced airflow [13]. However, Hemeon’s predicted results of induced airflow proved to be rough. Plinke proposed the conversion coefficient of induced airflow energy based on Hemeon’s research [14]. The experiment of the free-falling of material by Wypych and Cooper helped to determine the influences of induced airflow and dust emission during the free fall, respectively be the fall height and material temperature and Hemeon’s calculation formula of induced airflow is educed [15, 16]. Based on this work, Wheeler proposed a mathematical equation of dust generation rate under the influences of air humidity, falling height, and material feeding speed [17, 18]. Besides, Ansart analyzed the flow characteristics of free-falling plume and observed the influences on the plume with different outlet shapes. This approach was further used to quantify the movement of plume [19]. Based on the theory of hydrodynamics, Liu compared the induced airflow with the theoretical induced airflow to determine the ratio coefficient α. Further, the relationship between the ratio coefficient α with particle size, true density, and cross-sectional shape of the material stream was also analyzed [20]. Based on the analogous simulation experiment, Li determined that the induced airflow has an exponential relationship with the chute dip angle and the material feeding speed [21, 22]. I. N. Logachev [23, 24] proposed a method for computing the flow rate of air entrained by dry loose material while accounting for its particle-size distribution. A differential equation is obtained for the air entrained by loose material.

Compared with the previous research methods and results, the existing empiric formula for the calculation of induced airflow contains a large error with industry practice. This method is unable to guide the safe production of industrial and mining enterprises. Moreover, the interactions among these factors restrict and affect the dynamic characteristics of induced airflow and dust particles at the transshipment point, which could not be depicted precisely.

In this study, a multifactorial testing and experimental system are constructed independently to overcome the above-mentioned shortcomings. Also, the effects of different variables such as the bunker height, the chute angle, the feeding speed, the coal granularity, and the lower belt speed on the dynamic characteristics of induced airflow and dust emissions are determined by using the Box-Behnken response surface method for experimental design. The primary and secondary relations and the mutual functions among the yield factors of various influences are analyzed, which helps to extend the existing research and provide a theoretical foundation to guide the dust control at the transshipment point.

## 2. Experimental set-up and system

### 2.1 Sample preparation

The raw coal sample used in this experiment is taken from Cuncaota mine No.2, Shendong coal Refco Group Ltd. The raw coal sample is turned into small particles by crushing, screening, and classification. The granularity scale of the particles is 10 mm~40 mm and the moisture content is 6%.

### 2.2 Experimental apparatus

**Fig 3:**
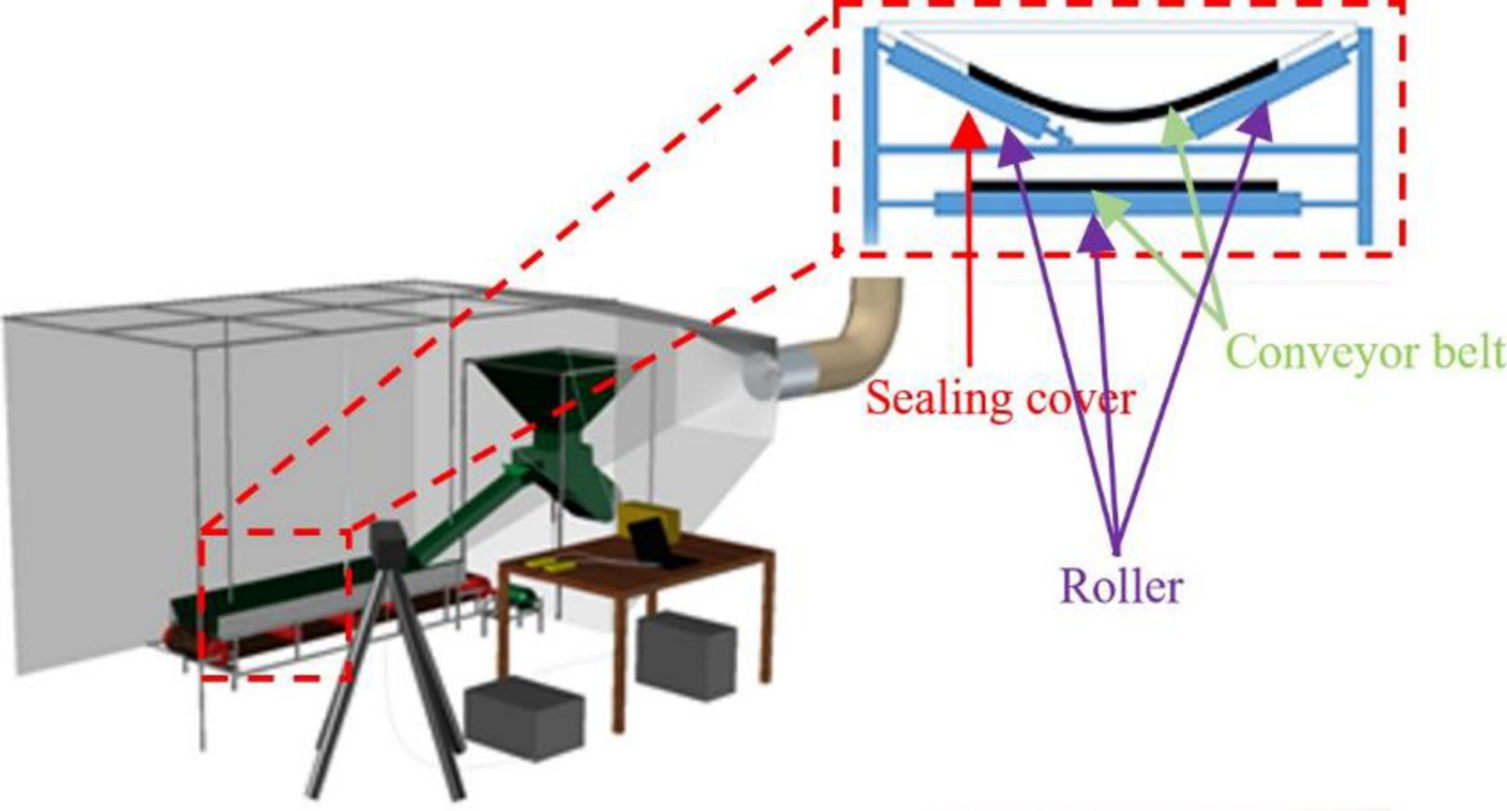
Schematic diagram of the experimental system.

The induced airflow and dust emission characteristics testing system is built, and its schematic diagram is demonstrated in Fig 3. The experimental system consists of an inverted cone hopper (JS type with a bucket capacity of 7.5t), frequency electro-vibrating feeder (GZ6S type with a range of 0~10 t/h), electric hoist (HXS-150F type with a lifting speed of 15 m/min), frequency belt conveyer (TD75 type with belt width of 500 mm and transmission speed with a range of 0~5 m/s), an online dust monitoring (JH-BF1000 type with a range of 0~1000 mg/m^3^), and a hot-wire anemometer (KIMO VT110 type with a range of 0~30 m/s). A 12 mm thickness polycarbonate hollow sheet is used to seal the skeletal structure of six surfaces and the polycarbonate board install in the belt, meanwhile, trepanning a holes on the surface of the polycarbonate board, the online dust monitoring and hot-wire anemometer was install through the measuring hole and the test position is 15cm away from the belt. This ensures that the experimental data error is less than 5%, and the online dust monitoring and hot-wire anemometer are immobilized to the square pipe steel section. The position of the measuring points in the experiment is arranged at 0.5m above the outlet.

### 2.3 Experimental procedures

**Fig 4:**
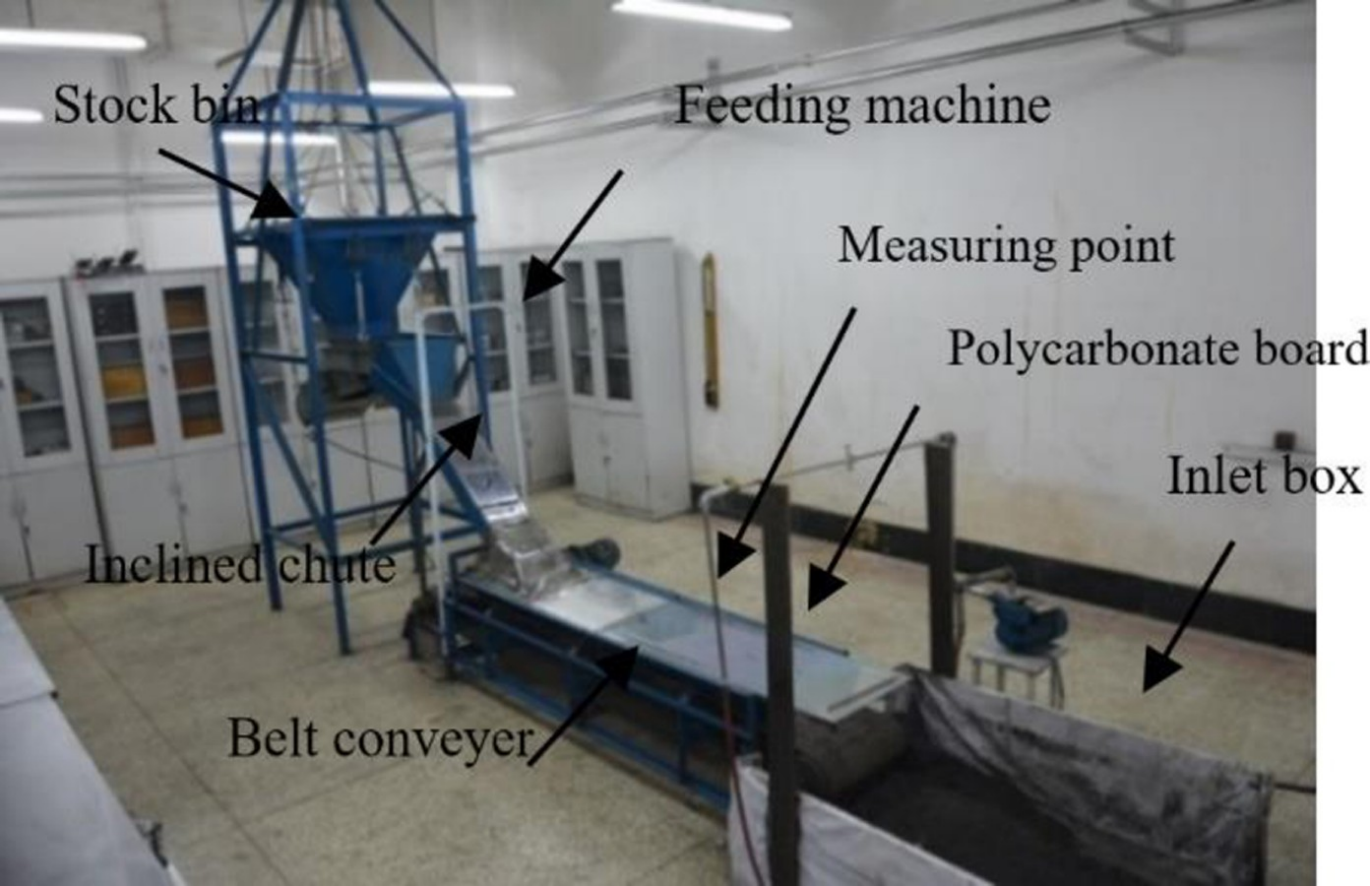
Panorama of the experimental system.

The induced airflow characteristics and dust particle diffusion influences the bunker height, chute angle, feeding speed, coal granularity, and belt speed. Based on the experimental system in Fig 4, 192 groups of experiments are studied. The specific test procedures are as follows: Various granularities of raw coal samples are put into the inverted cone hopper. This hopper is lifted at the height of 115cm, 95cm, and 75cm by an electric hoist. The chute angle is kept at 30°, 45° and, 60°, respectively. Meanwhile, the frequency belt conveyor speed is set to 0.31 m/s, 0.47 m/s, and 0.63 m/s, respectively, and the frequency electro-vibrating feeder is kept at 2t/h, 5t/h, and 8t/h, respectively. Before the formal start of the test, we conducted a series of trial tests and found that the difference between the data collected by the anemometer and dust concentration tester at adjacent time points was not more than 0.05m/s and 0.01mg/m3 respectively after the system started 50s. Therefore, we believe that a stable state has been reached inside the system at this time. The measured data can be used in research.

### 2.4 Experimental design and result

Dust diffusion characteristic is typically an interaction process of nonlinearity, nonequilibrium, inhomogeneous, and multiscale existing between the air and dust particles when induced airflow carries a large amount of dust particles to move together during the process of coal transshipment. Considering the results of the single-factor experiment, influences on induced airflow and dust diffusion characteristics are not only isolated among the factors such as bunker height, the chute angle, the feeding speed, the coal granularity, and the lower belt speed, but also the synergistic action between each other. Using the Box-Behnken response surface, a further study about the influence law can be advanced. Table 1 highlights the experimental influence factors and level setting, and the corresponding experimental schemes and results are enlisted in Table 2.

**Table 1:** Experimental factors and level.

Independent variable	Levels		
	-1	0	1
*X*_1_ = Bunker height (m)	0.75	0.95	1.15
*X*_2_ = Chute angle (°)	30	45	60
*X*_3_ = Feeding speed (t·h^–1^)	2	5	8
*X*_4_ = Coal granularity (mm)	10	25	40
*X*_5_ = Belt speed (m·s^–1^)	0.31	0.47	0.63
Dependent variable	Constraint		
*Y*_1_ = Induced airflow velocity (m·s^–1^)	--		
*Y*_2_ = Dust concentration (mg·m^–3^)	--		

**Table 2:** Box-Behnken design and experimental values of induced airflow velocity (*Y*_1_) and dust concentration (*Y*_2_).

Number	Independent variables					Response		Number	Independent variable					Response	
	*X*_1_	*X*_2_	*X*_3_	*X*_4_	*X*_5_	*Y*_1_	*Y*_2_		*X*_1_	*X*_2_	*X*_3_	*X*_4_	*X*_5_	*Y*_1_	*Y*_2_
1	1.15	45	8	25	0.47	1.675	1848.06	24	1.15	45	2	10	0.63	1.334	1527.12
2	1.15	30	8	25	0.63	1.603	1792.28	25	1.15	45	5	40	0.47	1.121	1540.08
3	1.15	45	5	25	0.63	1.574	1752.63	26	1.15	60	5	10	0.79	1.831	1939.83
4	1.15	60	8	25	0.63	1.869	1968.75	27	1.15	45	2	40	0.79	0.927	1327.04
5	1.35	45	2	25	0.63	1.543	1704.27	28	0.95	30	5	25	0.63	1.114	1269.17
6	1.15	45	5	40	0.79	1.221	1871.48	29	1.15	45	8	25	0.79	1.797	1934.79
7	1.35	45	5	25	0.79	1.846	1978.75	30	1.15	45	8	10	0.47	1.779	1934.92
8	1.15	45	5	10	0.47	1.598	1785.24	31	1.15	60	5	10	0.63	1.789	1905.64
9	1.15	60	2	25	0.63	1.341	1509.65	32	1.15	45	8	10	0.63	1.863	1998.96
10	0.95	45	5	25	0.79	1.281	1389.12	33	1.15	45	2	25	0.47	1.175	1403.89
11	1.15	45	2	10	0.79	1.371	1557.67	34	1.15	30	5	25	0.79	1.466	1679.71
12	1.35	45	5	25	0.47	1.722	1891.23	35	1.15	30	5	10	0.63	1.526	1729.38
13	1.15	45	2	25	0.79	1.279	1479.81	36	1.15	30	2	25	0.63	1.114	1355.56
14	1.35	45	5	10	0.63	1.912	2033.64	37	0.95	60	5	25	0.63	1.344	1417.75
15	1.15	45	2	40	0.63	0.899	1299.48	38	1.15	60	5	40	0.63	1.278	1650.71
16	1.15	45	8	40	0.63	1.337	1727.52	39	1.15	30	5	25	0.47	1.354	1597.42
17	1.35	45	5	40	0.63	1.377	1768.84	40	1.15	45	5	10	0.79	1.718	1619.57
18	1.15	60	5	25	0.79	1.725	1852.83	41	1.15	30	5	40	0.63	1.064	1488.93
19	0.95	45	5	25	0.47	1.176	1316.03	42	0.95	45	2	25	0.63	0.953	1093.45
20	1.35	45	8	25	0.63	1.993	2097.31	43	1.35	30	5	40	0.47	1.1176	1615.9
21	0.95	45	5	40	0.63	0.907	1214.17	44	0.95	45	8	25	0.63	1.409	1488.51
22	1.35	30	5	25	0.63	1.649	1834.92	45	0.95	45	5	10	0.63	1.337	1435.24
23	1.35	60	5	25	0.63	1.198	2012.95	46	1.15	60	5	25	0.47	2.266	2295.38

## 3. Results and discussion

### 3.1 Experimental analysis

**Fig 5:**
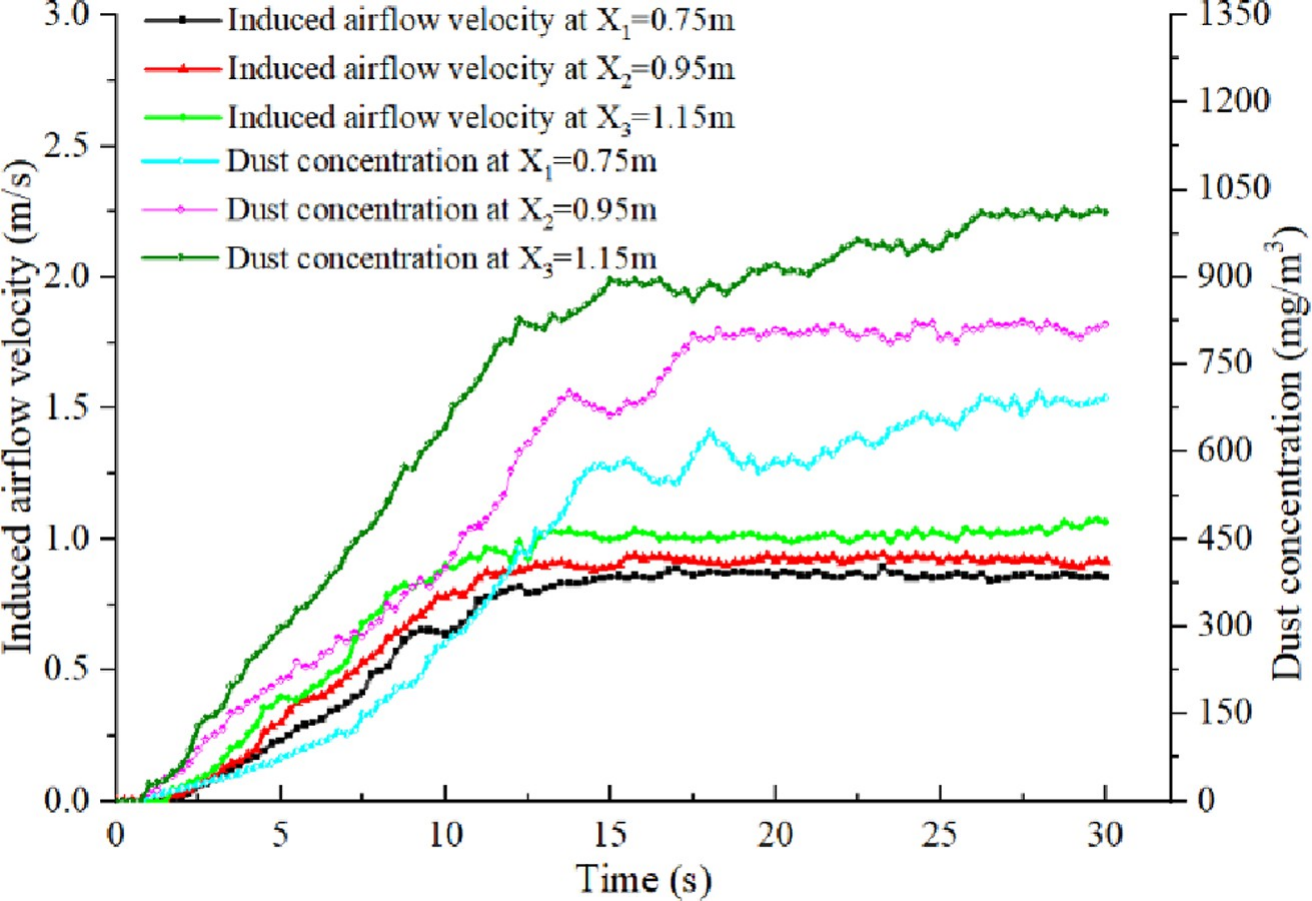
Induced airflow velocity and dust concentration with different bunker height versus time.

**Fig 6:**
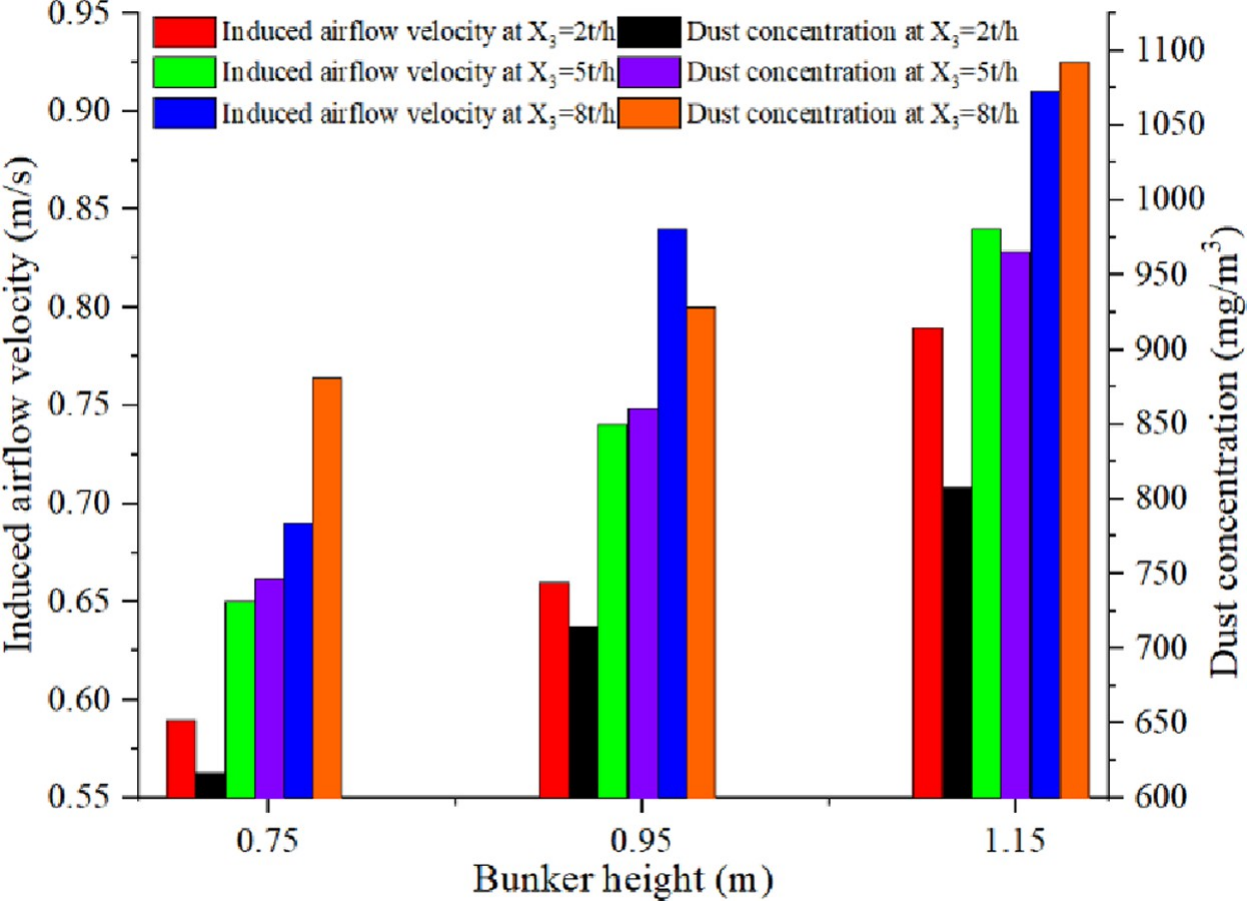
Induced airflow characteristics and dust emission with different bunker height and feeding speed.

As shown in Figs 5 and 6, since the bunker height increases from 0.75 m to 1.15 m, the maximum increment of the induced airflow velocity at the outlet of the guide chute is observed to be 0.35 m/s. This is because the coal potential energy and the final velocity of the falling coal increase with the increase in the height difference of the falling material. This leads to the increase in volume and the velocity of the induced airflow. This phenomenon is consistent with the research done by Professor Peter Wypych of the University of Wollongong [15]. The dust particles diffused for the first time under the negative pressure of the induced air flow and diffused for the second time with the direction of induced airflow on the belt vary instantly. The dust particles diffused in the guide chute move with the air flow and are eventually discharged into the space outside the guide chute. This leads to the rise in serious dust pollution outside the guide chute.

With the increase in the feed speed from 2t/h to 8t/h, the increment of the induced airflow velocity at the outlet of the guide chute is recorded to be 51%. When the feed quantity and the blanking height difference increase simultaneously, the effect of coal on the drag force of air flow increases with the increase of the amount of dispersed coal in the periphery of the feed material flow. Since the blanking process is always in a large acceleration state, the amount of air ensnared by the particle flow increases, and the disturbance of particles to the air strengthens. This leads to the increase in the velocity difference with the increase in the height difference of blanking.

### 3.2 Response value experimental results statistical analysis

Analysis of variance (ANOVA) is applied to the obtained responses. Table 3 enlists the experimental results’ statistical analysis of response values *Y*_1_ and *Y*_2_ with different fitted models. The F value is the statistic value of the F test. F test is a test that statistical values obey f-distribution under null hypothesis. It is usually used to analyze statistical models with more than one parameter to determine whether all or part of the parameters in the model are suitable for estimating the matrix. Prob > F is the P value. If P≤0.05, it indicates that the item has significant influence on Y. If P≤0.01, it indicates that the item has extremely significant influence on Y. If P > 0.05, it indicates that the item has no significant influence on Y.

**Table 3:** ANOVA analysis results for response.

Model	*F* value		Prob>F		Adjusted R-Squared		Predicted R-Squared			
	*Y*_1_	*Y*_2_	*Y*_1_	*Y*_2_	*Y*_1_	*Y*_2_	*Y*_1_	*Y*_2_	*Y*_1_	*Y*_2_
Linear	3.27	10.36	0.3218	<0.0001	0.9139	0.8868	0.8731	0.8323		Suggested
2FI	8.83	5.88	<0.0001	0.0121	0.9596	0.9463	0.8543	0.8356	Suggested	Suggested
Quadratic	2.32	2.51	0.0731	0.0569	0.9336	0.9258	0.8582	0.8164		
Cubic	0.85	22.21	0.6441	0.014	0.8913	0.9192	0.7770	0.4229		

The two-factor interactive model value *F* of the response value *Y*_1_ is maximum while the Prob>value F is minimum. This indicates that the significance of this fitted model experimental data. The fitting effect of response value *Y*_2_ with the linear model is better than that of the other models. Design-Expert software suggests a linear model and two-factor interactive model, out of which the two-factor interactive model of the relatively high-order moment has been chosen. The two-factor interactive model’s coefficient of determination of response values *Y*_1_ and *Y*_2_ are 0.9736 and 0.9563, respectively. Both these values are greater than 0.8. The two-factor interactive model’s adjustment coefficient of determination of response values *Y*_1_ and *Y*_2_ are 0.9596 and 0.9463, respectively, and the predictive coefficient of determination is 0.9393 and 0.9345, respectively. The D-values are 0.0203 and 0.0118, which are both less than 0.2. This suggests that the two-factor interactive model has a high correlation with the experiment. Above all, the response values *Y*_1_ and *Y*_2_ both adopt the two-factor interactive model in this paper.

### 3.3 Coupling effect model building

Table 4 highlights the analytical results of confidence level about all the influent factors of response values *Y*_1_ and *Y*_2_ in the two-factor interactive model by Design–Expert.

**Table 4:** Parameter’s estimation of the model equation.

Intercept	Coefficient estimate		Standard error		95% Confidence interval low		95% Confidence interval high		*p*-value Prob>F	
	*Y*_1_	*Y*_2_	*Y*_1_	*Y*_2_	*Y*_1_	*Y*_2_	*Y*_1_	*Y*_2_	*Y*_1_	*Y*_2_
constant	1.43	7.39	0.018	3.10 × 10^−3^	1.39	7.38	1.47	7.41	-	-
*X*_1_	0.22	0.16	5.59 × 10^−3^	5.05 × 10^−3^	0.17	016	0.28	0.21	<0.0001	<0.0001
*X*_2_	0.25	0.085	5.31 × 10^−3^	4.79 × 10^−3^	0.19	0.11	0.30	0.16	0.0174	0.0134
*X*_3_	0.075	0.13	5.43 × 10^−3^	4.92 × 10^−3^	0.014	0.024	0.14	0.074	<0.0001	<0.0001
*X*_4_	-0.26	-0.093	5.01 × 10^−3^	4.60 × 10^−3^	-0.30	-0.087	-0.19	-0.042	<0.0001	0.0018
*X*_5_	0.073	0.055	5.12 × 10^−3^	4.63 × 10^−3^	0.016	8.66 × 10^−3^	0.13	0.055	0.0130	0.0353
*X*_1_ *X*_2_	-0.14	0.08	0.011	0.01	-0.12	-0.078	0.11	0.017	0.9278	0.5072
*X*_1_ *X*_3_	-0.0051	-0.041	0.011	9.72 × 10^−3^	-0.25	-0.049	-0.032	0.043	0.0129	0.1811
*X*_1_ *X*_4_	-0.054	-0.078	0.011	9.72 × 10^−3^	-0.16	-0.040	0.056	0.051	0.3220	0.1943
*X*_1_ *X*_5_	0.033	0.082	0.011	9.72 × 10^−3^	-0.077	-0.046	0.14	0.045	0.5481	0.1690
*X*_2_ *X*_3_	0.0063	-0.019	0.011	0.01	-0.11	-0.056	0.12	0.039	0.9104	0.7543
*X*_2_ *X*_4_	0.018	0.069	9.51 × 10^−3^	8.55 × 10^−3^	-0.10	-9.23 × 10^−3^	0.087	0.070	0.8658	0.3123
*X*_2_ *X*_5_	0.02	-0.073	9.72 × 10^−3^	8.06 × 10^−3^	-0.048	-0.032	0.14	0.044	0.3347	0.8337
*X*_3_ *X*_4_	-0.0079	0.052	0.01	9.23 × 10^−3^	-0.089	-0.045	0.12	0.044	0.7328	0.2343
*X*_3_ *X*_5_	0.044	0.01	0.01	9.23 × 10^−3^	-0.10	-0.029	0.14	0.072	0.7379	0.2699
*X*_4_ *X*_5_	0.014	0.11	9.01 × 10^−3^	8.06 × 10^−3^	-0.078	0.012	0.11	0.089	0.7581	0.0357

The order of influent factors on the response values can be determined based on the estimated absolute value of the two-factor interactive model equation parameter. With the interaction of factors, the order of influent factors about the induced airflow velocity is coal cinder granularity *X*_4_(*p*<0.0001)>material dropping amount *X*_3_(*p*<0.0001)>material dropping distance *X*_1_(*p*<0.0001)>chute angle *X*_2_(*p*<0.05)>belt conveyor velocity *X*_5_(*p*<0.05). The order of influent factors about the dust concentration is material dropping distance *X*_1_(*p*<0.0001)>material dropping amount *X*_3_(*p*<0.0001)>coal cinder granularity *X*_4_(*p*<0.0001)>chute angle *X*_2_(*p*<0.05)>belt conveyor velocity *X*_5_(*p*<0.05).

The relationship between influent factors and response values are described by the two-factor interactive model equation parameter. The corresponding regression model is:

*Y*_1_ = 1.43+0.22*X*_1_+0.075*X*_2_+0.25*X*_3−_0.26*X*_4_+0.073*X*_5_-0.14*X*_1_*X*_2_−0.0051*X*_1_*X*_3_−0.054*X*_1_*X*_4_+0.033*X*_1_*X*_5_+0.0063*X*_2_*X*_3_+0.018*X*_2_*X*_4_+0.02*X*_2_*X*_5_−0.0079*X*_3_*X*_4_+0.044*X*_3_*X*_5_+0.014*X*_4_*X*_5_

*Y*_2_ = 7.39+0.16*X*_1_+0.085*X*_2_+0.13*X*_3_−0.093*X*_4_+0.055*X*_5_+0.08*X*_1_*X*_2_−0.041*X*_1_*X*_3_−0.078*X*_1_*X*_4_+0.082*X*_1_*X*_5_−0.019*X*_2_*X*_3_+0.069*X*_2_*X*_4_−0.073*X*_2_*X*_5_+0.052*X*_3_*X*_4_+0.01*X*_3_*X*_5_+0.11*X*_4_*X*_5_

**Fig 7:**
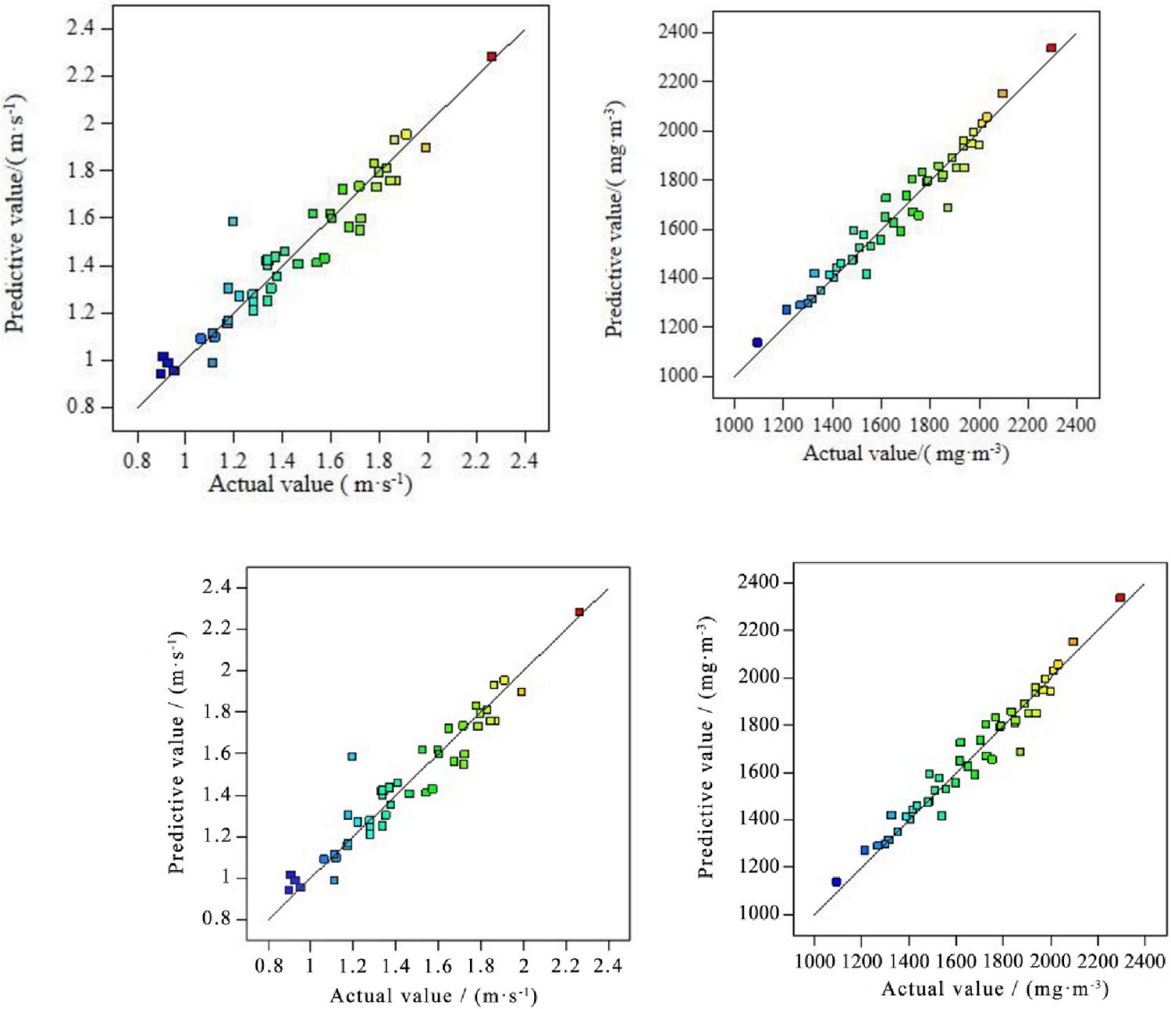
Predicted vs. actual of internally studentized residuals.

Fig 7 demonstrates the relation between experimental results about two kinds of response values of induced airflow and dust concentration and the predictive value of the two-factor interactive model. This suggests that the distribution of predictive value data points is not only symmetrical but also approaching the diagonal line, indicating the reliability of the two-factor interactive model prediction.

### 3.4 Response surface analysis

Experimental design and data are used by the response surface methodology to construct a three-dimensional graph or a two-dimensional contour plot. It adopts a multivariate quadratic regression equation to fit functional relation between factors and response values and reflects the influences of response values on various factors.

#### 3.4.1 Multiple factors affecting the induced airflow velocity (*Y*_1_)

**Fig 8:**
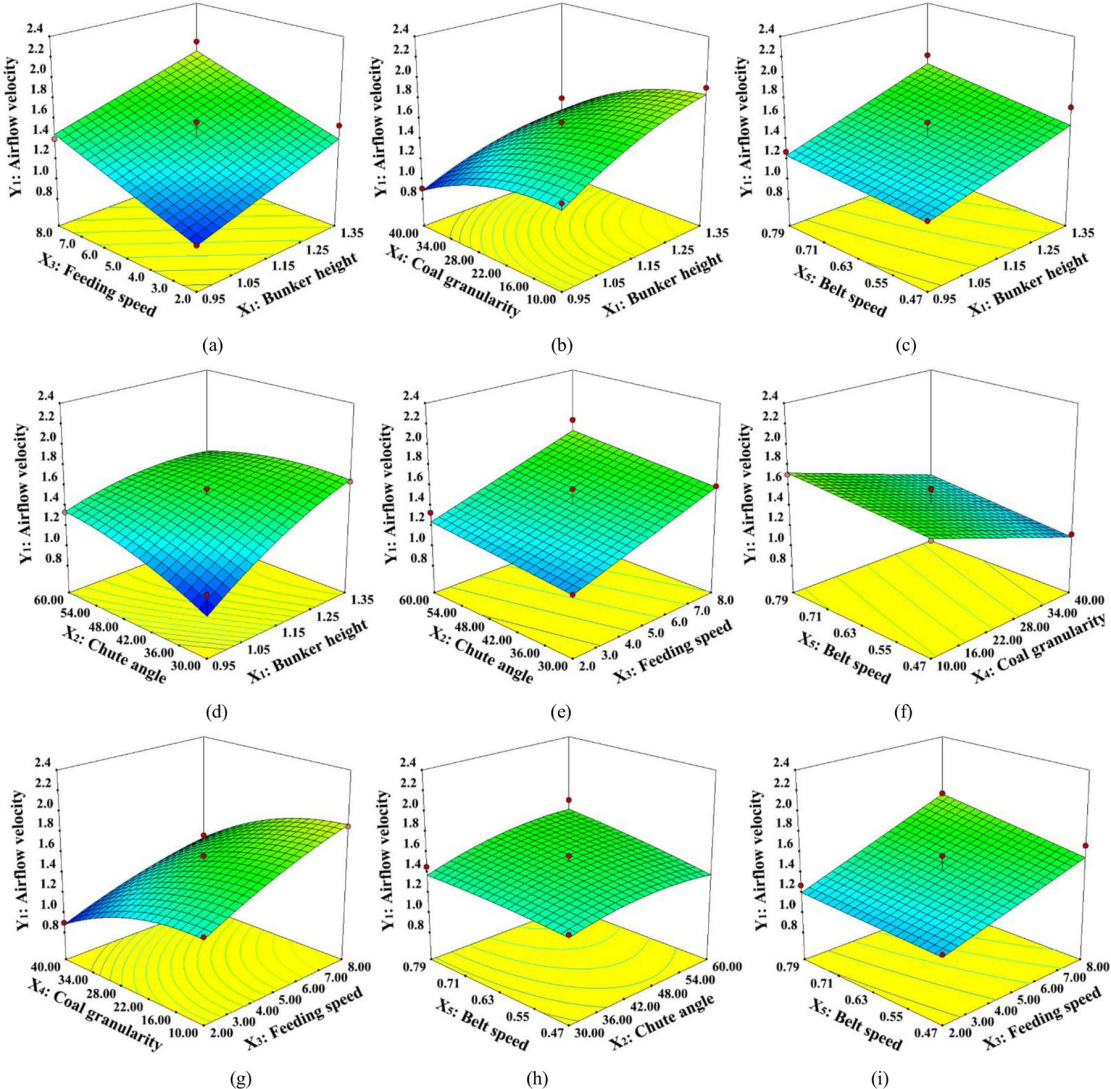
Effect of interaction between factors on the induced airflow velocity.

During the transshipment of coal, the ambient air is dragged and squeezed to produce high-pressure induced airflow. Fig 8 depicts the three-dimensional response surface of induced airflow velocity with interaction among factors.

As demonstrated in Fig 8B, the three-dimensional surface contour line of sample granularity and material dropping amount is densely distributed. This implies the significant interaction of the two factors. In fact, with the increase of mass flow of large particle samples through the chute in unit time, more energy can be transferred to the induced airflow under identical operating conditions.

As for the abscissa axis, in the Fig 8D, the three-dimensional surface contour line of silo height and chute angle is densely distributed, which implies that the silo height has a more remarkable influence on the induced airflow velocity than the chute angle. There is a huge pressure difference between the upper and the lower surface due to the increase of drop height. Besides, with the joint effect of shear force on the material surface, more air is inhaled into the chute, and the induced airflow velocity keeps increasing. But in the Fig 8E, we can found that three-dimensional surface contour line of the lower belt running velocity and chute angle shows almost no distortion, which suggests little interaction between the two factors. The effective cross-sectional area of airflow is reduced due to the stacking thickness on the belt surface influenced by a lower belt running velocity. This has an indirect influence on the chute’s internal pressure.

#### 3.4.2 Multiple factors affecting dust concentration (*Y*_*2*_)

Dust particles entrained by the induced airflow caused by the self-motion of materials are diffused with the effect of the negative pressure. Besides, the induced airflow direction of the belt changes at every short interval, which lets the entrained dust particles to re-diffuse. Fig 7 highlights the three-dimensional response surface of dust concentration with interaction among the factors.

**Fig 9:**
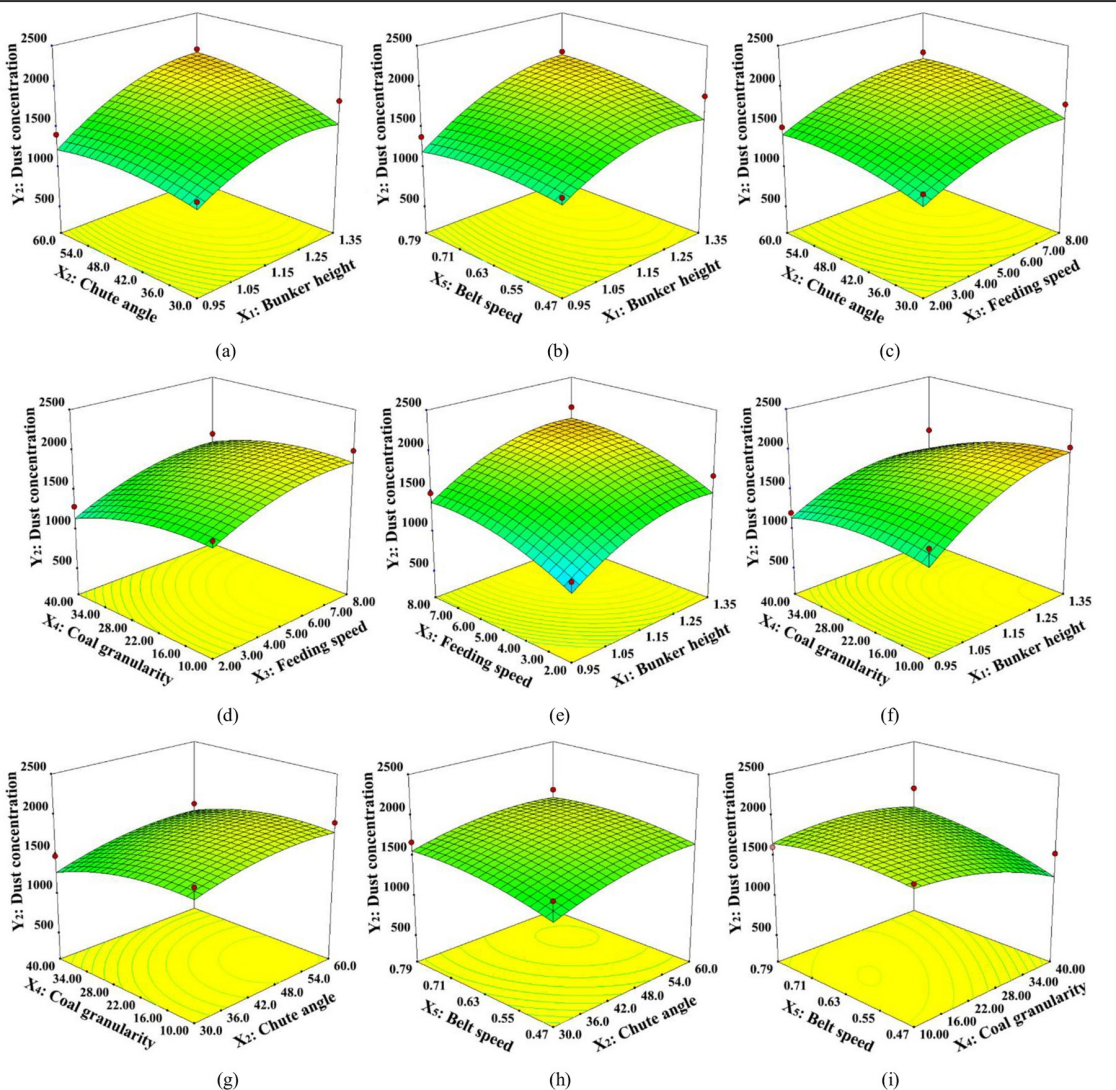
Effect of interaction between factors on dust concentration.

As displayed in Fig 9, when the other four factors are at their selected conditional level, the three-dimensional surface contour line of dust concentration and silo height are densely distributed and steeply curve. This indicates the significant effect of the silo height on the dust concentration. This diagram is in accordance with the analysis of model variance. In fact, in the entire process of dropping impact, due to the reduction of vertical static pressure and spontaneous turbulence, the induced airflow keeps entraining fresh air to form eddy current. Besides, the dust particles lead to an irregular diffusion with the effect of eddy current.

As for the abscissa axis, in the Fig 9D, the three-dimensional surface contour line of sample granularity and material dropping amount is more densely distributed. This illustrates that the material dropping amount has a more remarkable influence on the dust concentration than the sample’s granularity. Meanwhile, under the conditions of multiple factors and single factors, the dust concentration disaccords with the changing regularity of sample granularity. This implies that there exists an obvious interaction among the factors. With the decrease of sample granularity, the voidage of material heaped on the chute surface reduces. This leads to the reduction of the volume of induced airflow. But in the Fig 9C and 9J, three-dimensional surface contour line of the lower belt running velocity, sample granularity, and chute angle demonstrates almost no distortion, suggesting a little interaction among the factors. Due to the traction airflow formed around the belt conveyor in the process of lower belt operation, a large amount of dust particles arise at the moment of material impacting on belt diffuses along the direction of the belt moving by changing the internal pressure of the chute. However, since the traction airflow is just around the belt surface, it has little contribution to the dust concentration.

### 3.5 The comparative analysis of experimental results and theoretical arithmetic

Currently, scholars all over the world have derived some empirical formulas for the induced airspeed under different assumptions, which primarily include:

More recently, Hemeon and Morrison had a theoretical study on the induced airflow by the resistance coefficient formula. They provided a semi-empirical formula:

$$v={\left(\frac{0.66gm_{p}L^{2}}{Ad_{p}\rho_{p}}\right)}^{1/3}$$ (1)

In the above equation, *v* indicates the induced airflow velocity, *m*_*p*_ suggests the mass flow rate of materials, *g* denotes the gravitational acceleration, *d*_*p*_ is the diameter of coal sample, *ρ*_*p*_ represents the density of coal, *L* indicates the chute length, and *A* suggests the cross-sectional area.

LI Xiaochuan identified the influence regulars of a single factor on the induced airflow, and suggested a similar criterion number of the induced airflow. He also promoted the calculation formula of the induced airflow velocity as:

$$v=\frac{k}{d_{p}\rho_{p}}{\left(\frac{m_{p}}{\rho_{g}g^{0.5}h^{1.5}D}\right)}^{0.27}$$ (2)

In the above equation, *k* indicates the non-dimensional coefficient, *ρ*_*g*_ represents the air density, and *D* denotes the bottom diameter of the funnel.

**Fig 10:**
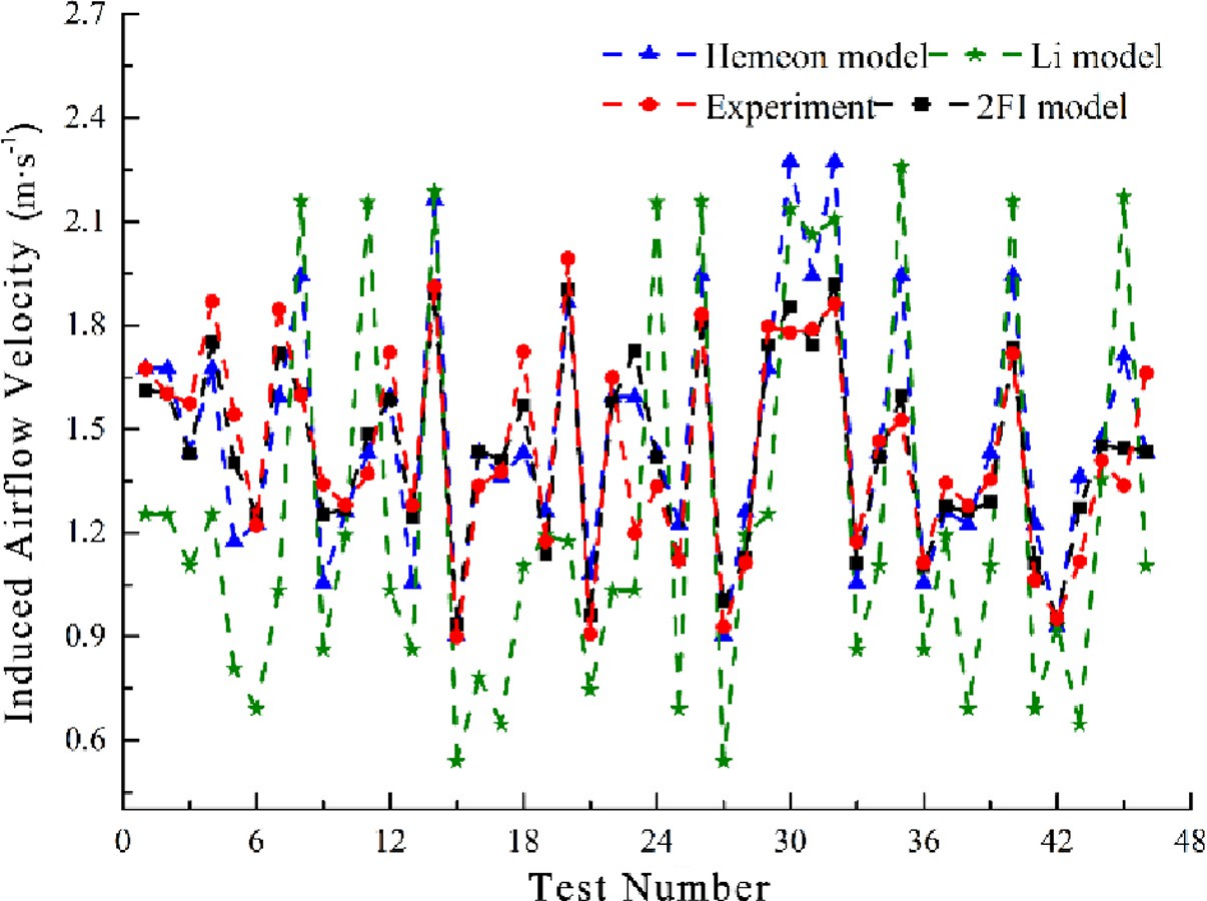
The theoretical curve and measured curve correlation of induced airflow velocity.

**Fig 11:**
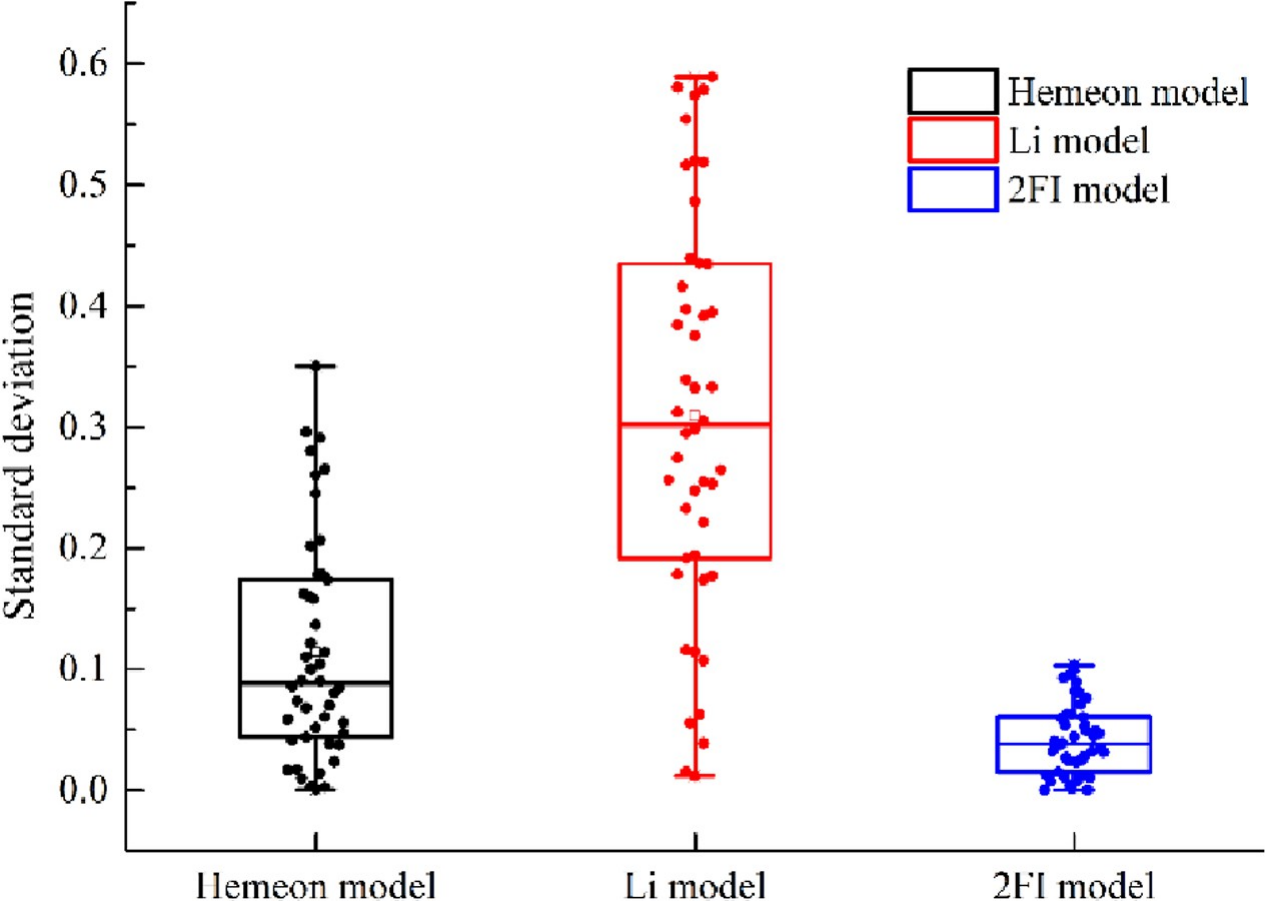
Standard deviation of induced airflow velocity box and whiskers.

Concerning various experimental schemes and results and then putting a comparative analysis into the two-factor interactive model of induced airflow velocity and the model of Hemeon, Li, Fig 10 illustrates the variations of induced airflow velocity under different experimental schemes, and Fig 11 demonstrates the induced airflow velocity error bar of different calculation models.

According to Figs 10 and 11;

1. When the maximum experimental measured value of induced airflow velocity is 1.993 m/s, the theoretical value of induced airflow velocity of Hemeon, Li model, and two-factor interactive model are 1.865 m/s,1.174 m/s, and1.904 m/s, respectively, and the corresponding error values are 0.128 m/s, 0.819 m/s, and 0.089 m/s.
2. As compared to the Hemeon and Li calculation model, the two-factor interactive model influences the interactive effect among the factors into consideration, whose error range of theoretical calculation results and experimentally measured values is 0.0009 m/s ~ 0.116 m/s. The importance of interactive effect among the factors is illustrated by a good consistency, which can accurately describe the characteristic of induced airflow in the transshipment point with different operational conditions.

## 4. Field measurement

The induced air flow velocity and dust concentration at 10 transfer points of Cuncaota No. 2 mine of Shendong Coal Group are measured on-site to verify the accuracy of the two-factor interaction model equation. The 10 transfer points are, respectively: ① transfer point at the upper warehouse compartment 1; ② transfer point at the upper warehouse compartment 2; ③ transfer point at the head of the main shaft; ④ transfer point at the coal mine roadway 22; ⑤ transfer point at the coal mine roadway 33; ⑥ transfer point at the head of conveyor belt in the main crossheading 22109; ⑦ transfer point at the head of conveyor belt in the main crossheading 22121; ⑧ transfer point at the head of conveyor belt in the main crossheading 31204; ⑨ transfer point at the head of conveyor belt in the subsidiary crossheading 31205; ⑩ transfer point at the head of conveyor belt in fully-mechanized face 22121.

### 4.1 Setting of measuring points

The test points are arranged at 0.5 m above the outlet of the guide trough at the transfer point according to the actual situation of each transfer point in Cuncaota No. 2 Mine. ZRQFF30 intelligent hot-wire anemometer and HMFC-ZDB explosion-proof dust tester are used to measure the induced airspeed and dust concentration. Each test section is tested thrice and the average value is taken.

### 4.2 Analysis of measurement results

**Fig 12:**
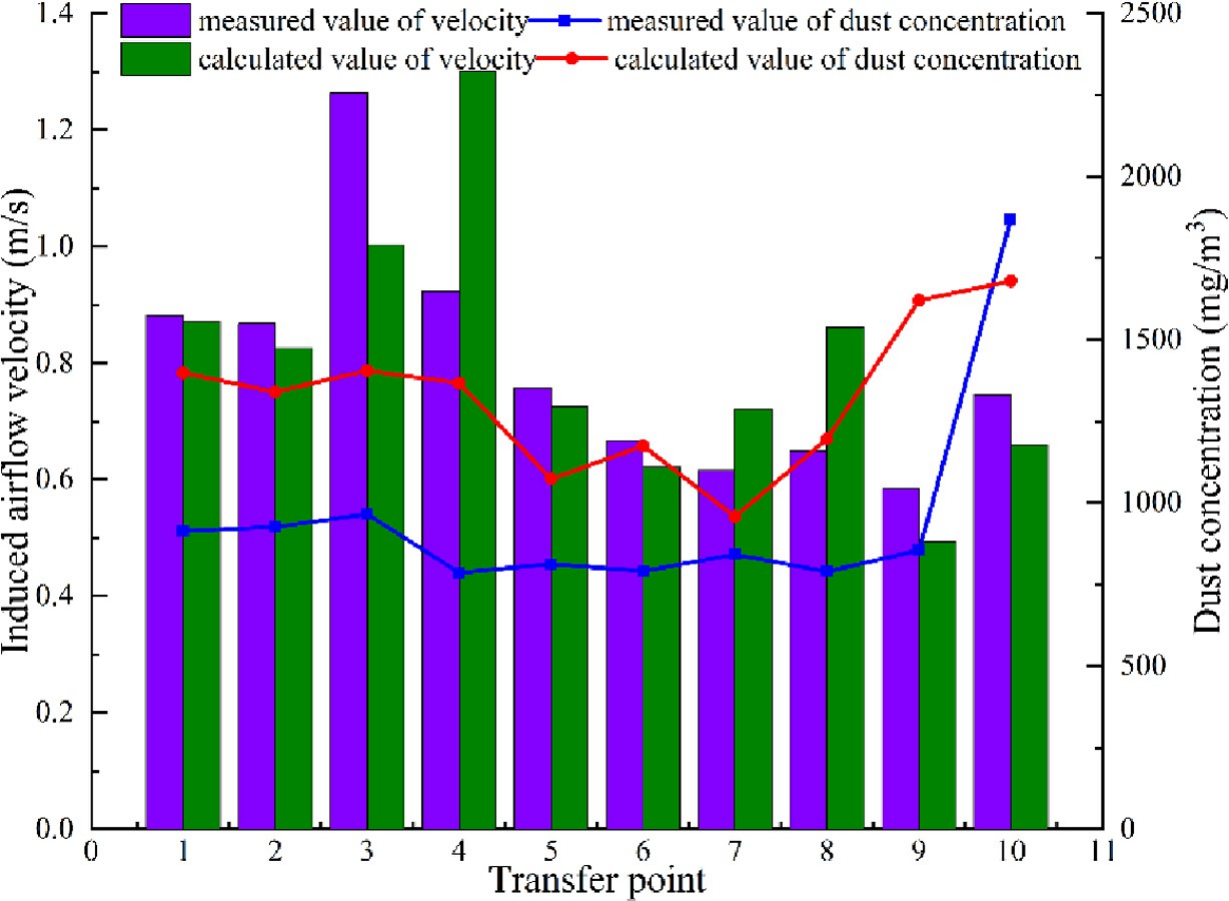
Measured and calculated values of induced air velocity and dust concentration at the transfer point.

Table 5 enlists the Dimensions and operation parameters of each transfer point, and the Fig 12 show the measured value and the theoretically calculated values of the induced air velocity and dust concentration at each transfer point of Cuncaota No. 2 mine, respectively.

**Table 5:** The dimensions and operation parameters of each transfer point.

Transfer point	Bunker height (m)	Chute angle(°)	Feeding speed ((t·h^–1^)	Coal granularity (mm)	Belt speed(m·s^–1^)
①	4.78	45	1000	50	4.5
②	4.17	65	800	50	4.5
③	3.51	90	1500	50	4
④	5.98	88	1200	50	5
⑤	4.61	60	1000	50	4.5
⑥	2.91	48	800	50	4
⑦	4.59	78	600	50	5
⑧	3.01	80	800	50	4
⑨	3.24	40	1100	50	4
⑩	4.46	35	800	50	4.5

It can be deduced from Fig 12 that the relative errors in the measured and calculated values of the induced air velocity and dust concentration at each point of Cuncaota No. 2 mine are marginal, and the relative errors range from 1.02% to 8.76% and 1.68% to 6.39%, respectively. This indicates that the two-factor interaction model can accurately predict the induced air velocity and dust concentration at the transfer point.

## 5. Conclusion

1. The two-factor interactive model of response value is established by aiming at the influent regularity of the induced airflow velocity and dust diffusion characteristics, influenced by 5 factors: silo height, material dropping amount, chute angle, coal granularity, and lower belt velocity. This is done using Box-Behnken response surface design. The error scale of the model is as low as 5%. This model can be used to analyze the interaction effects among the factors and to carry out optimization and collocation of factors.
2. Three-dimensional surface contour line of sample granularity and material dropping amount is densely distributed, and the coal granularity has the most significant effect on the induced airflow velocity. In fact, when the other four factors are at their selected conditional level, the three-dimensional surface contour line of dust concentration and silo height seems densely distributed and steeply curve. Silo height has the most significant effect on dust concentration. Lower belt velocity has less influence on the induced airflow velocity and dust concentration.
3. Under the condition of multiple factors and single factor, dust concentration disaccords with the change in the regularity of the sample granularity. Since the effect of factors on the induced airflow is not isolated, it stays connected and restrained to each other. As the sample granularity reduces, the voidage of material heaped on the chute surface reduces, but the material dropping amount rises. This leads to the reduction of volume of induced airflow. This is the essential reason for the existing complexity in the induced airflow characteristic and dust diffusion with interactive effect among factors during the transshipment.

## Supporting information

S1 Data All the data is in a file called supporting information. (DOCX)
