# Supplementary material for: Study on the characteristics of induced airflow and particle dispersion based on the multivariate two-factor model
Source: PLoS One. 2022 Feb 8;17(2):e0263740. doi: 10.1371/journal.pone.0263740 (PMC8824373; doi:10.1371/journal.pone.0263740)
Supplement: S1 Data — (DOCX) [file pone.0263740.s001.docx]

| Fig. 1. National occupational disease report ofin 2011~2017 | | | |
| --- | --- | --- | --- |
| Year | Pneumoconiosis | Pneumoconiosis | Coal miners pneumoconiosis percent |
|  | Number | Number | % |
| 2011 | 26401 | 29879 | 51.61 |
| 2012 | 24206 | 27420 | 48.87 |
| 2013 | 23152 | 26393 | 57.13 |
| 2014 | 26873 | 29972 | 62.52 |
| 2015 | 26081 | 29180 | 61.03 |
| 2016 | 28088 | 31789 | 66.09 |
| 2017 | 22790 | 26756 | 72.66 |

Figures 2 and 3 and 4 do not require data

Fig. 5. Induced airflow velocity and dust concentration with different bunker height versus time

| Time | Induced airflow velocity | Induced airflow velocity | Induced airflow velocity | Dust concentration |
| --- | --- | --- | --- | --- |
| s | m/s | m/s | m/s | mg/m^3^ |
|  | Induced airflow velocity at X_1_=0.75m | Induced airflow velocity at X_2_=0.95m | Induced airflow velocity at X_3_=1.15m | Dust concentration at X_1_=0.75m |
| 0 | 0 | 0 | 0 | 0 |
| 0.25 | 0 | 0 | 0 | 0 |
| 0.5 | 0 | 0 | 0 | 0 |
| 0.75 | 0 | 0 | 0 | 0 |
| 1 | 0 | 0 | 0 | 8.99 |
| 1.25 | 0 | 0 | 0 | 10.2 |
| 1.5 | 0 | 0 | 0 | 14.2 |
| 1.75 | 0.008 | 0.021 | 0.041 | 17.5 |
| 2 | 0.012 | 0.028 | 0.051 | 21.6 |
| 2.25 | 0.031 | 0.041 | 0.067 | 24.6 |
| 2.5 | 0.056 | 0.065 | 0.084 | 28.4 |
| 2.75 | 0.065 | 0.081 | 0.097 | 30.2 |
| 3 | 0.082 | 0.098 | 0.123 | 37.5 |
| 3.25 | 0.095 | 0.124 | 0.158 | 39.1 |
| 3.5 | 0.123 | 0.145 | 0.198 | 42.8 |
| 3.75 | 0.137 | 0.154 | 0.214 | 46.7 |
| 4 | 0.159 | 0.178 | 0.254 | 53.4 |
| 4.25 | 0.165 | 0.198 | 0.284 | 58.3 |
| 4.5 | 0.193 | 0.265 | 0.357 | 61.7 |
| 4.75 | 0.219 | 0.284 | 0.362 | 64.9 |
| 5 | 0.232 | 0.301 | 0.397 | 73.4 |
| 5.25 | 0.254 | 0.344 | 0.389 | 79.1 |
| 5.5 | 0.278 | 0.375 | 0.387 | 84.9 |
| 5.75 | 0.291 | 0.391 | 0.412 | 92.4 |
| 6 | 0.298 | 0.395 | 0.436 | 96.2 |
| 6.25 | 0.311 | 0.401 | 0.451 | 100.2 |
| 6.5 | 0.341 | 0.425 | 0.487 | 107.8 |
| 6.75 | 0.354 | 0.451 | 0.501 | 119.2 |
| 7 | 0.372 | 0.478 | 0.531 | 114.9 |
| 7.25 | 0.397 | 0.497 | 0.614 | 120.5 |
| 7.5 | 0.413 | 0.532 | 0.675 | 147.5 |
| 7.75 | 0.484 | 0.551 | 0.701 | 151.2 |
| 8 | 0.498 | 0.578 | 0.724 | 168.4 |
| 8.25 | 0.514 | 0.621 | 0.784 | 175.3 |
| 8.5 | 0.574 | 0.644 | 0.808 | 192.4 |
| 8.75 | 0.611 | 0.664 | 0.828 | 198.2 |
| 9 | 0.638 | 0.697 | 0.815 | 201.2 |
| 9.25 | 0.655 | 0.714 | 0.842 | 214.6 |
| 9.5 | 0.651 | 0.743 | 0.837 | 245.6 |
| 9.75 | 0.647 | 0.781 | 0.866 | 264.3 |
| 10 | 0.636 | 0.777 | 0.899 | 270.7 |
| 10.25 | 0.654 | 0.795 | 0.887 | 284.8 |
| 10.5 | 0.679 | 0.786 | 0.913 | 291.4 |
| 10.75 | 0.712 | 0.812 | 0.935 | 310.2 |
| 11 | 0.763 | 0.852 | 0.925 | 324.7 |
| 11.25 | 0.777 | 0.875 | 0.961 | 341.2 |
| 11.5 | 0.782 | 0.864 | 0.957 | 365.1 |
| 11.75 | 0.799 | 0.879 | 0.947 | 384.2 |
| 12 | 0.81 | 0.871 | 0.921 | 400.8 |
| 12.25 | 0.821 | 0.884 | 0.987 | 432.5 |
| 12.5 | 0.792 | 0.888 | 0.923 | 421.4 |
| 12.75 | 0.798 | 0.902 | 0.999 | 462.3 |
| 13 | 0.81 | 0.899 | 1.018 | 455.4 |
| 13.25 | 0.821 | 0.907 | 1.035 | 471.3 |
| 13.5 | 0.832 | 0.914 | 1.022 | 490.6 |
| 13.75 | 0.833 | 0.902 | 1.033 | 517.9 |
| 14 | 0.829 | 0.894 | 1.018 | 547.8 |
| 14.25 | 0.837 | 0.891 | 1.021 | 564.3 |
| 14.5 | 0.845 | 0.884 | 1.014 | 572.7 |
| 14.75 | 0.852 | 0.889 | 1.001 | 575.3 |
| 15 | 0.856 | 0.894 | 0.999 | 569.8 |
| 15.25 | 0.858 | 0.902 | 1.005 | 578.5 |
| 15.5 | 0.851 | 0.932 | 1.012 | 585.2 |
| 15.75 | 0.862 | 0.938 | 1.036 | 574.3 |
| 16 | 0.855 | 0.925 | 1.024 | 565.4 |
| 16.25 | 0.851 | 0.934 | 1.017 | 551.3 |
| 16.5 | 0.862 | 0.932 | 1.001 | 547.8 |
| 16.75 | 0.878 | 0.927 | 1.009 | 552.6 |
| 17 | 0.887 | 0.921 | 1.002 | 545.6 |
| 17.25 | 0.872 | 0.924 | 1.009 | 569.7 |
| 17.5 | 0.862 | 0.917 | 0.997 | 594.7 |
| 17.75 | 0.865 | 0.915 | 1.001 | 613.2 |
| 18 | 0.877 | 0.911 | 1.012 | 633.6 |
| 18.25 | 0.871 | 0.908 | 1.001 | 615.3 |
| 18.5 | 0.866 | 0.904 | 1.006 | 609.4 |
| 18.75 | 0.875 | 0.911 | 1.015 | 588.3 |
| 19 | 0.877 | 0.914 | 1.012 | 572.7 |
| 19.25 | 0.872 | 0.917 | 1.018 | 590.3 |
| 19.5 | 0.869 | 0.925 | 1.009 | 565.4 |
| 19.75 | 0.874 | 0.936 | 1.002 | 574.6 |
| 20 | 0.862 | 0.921 | 1.011 | 584.2 |
| 20.25 | 0.875 | 0.932 | 1.001 | 578.9 |
| 20.5 | 0.871 | 0.919 | 0.991 | 589.2 |
| 20.75 | 0.862 | 0.926 | 1.001 | 581.6 |
| 21 | 0.859 | 0.917 | 1.005 | 574.5 |
| 21.25 | 0.876 | 0.929 | 1.009 | 586.9 |
| 21.5 | 0.862 | 0.923 | 1.012 | 604.2 |
| 21.75 | 0.857 | 0.932 | 1.018 | 594.6 |
| 22 | 0.854 | 0.911 | 0.999 | 614.7 |
| 22.25 | 0.867 | 0.921 | 0.991 | 621.3 |
| 22.5 | 0.871 | 0.927 | 1.005 | 627.9 |
| 22.75 | 0.857 | 0.939 | 1.018 | 615.3 |
| 23 | 0.854 | 0.932 | 1.007 | 610.4 |
| 23.25 | 0.895 | 0.943 | 1.021 | 619.8 |
| 23.5 | 0.868 | 0.918 | 0.991 | 637.8 |
| 23.75 | 0.872 | 0.935 | 1.024 | 641.2 |
| 24 | 0.853 | 0.924 | 1.009 | 647.9 |
| 24.25 | 0.851 | 0.939 | 1.035 | 655.9 |
| 24.5 | 0.861 | 0.929 | 1.011 | 664.3 |
| 24.75 | 0.853 | 0.934 | 1.027 | 651.3 |
| 25 | 0.854 | 0.914 | 1.019 | 657.4 |
| 25.25 | 0.859 | 0.927 | 1.011 | 650.2 |
| 25.5 | 0.868 | 0.918 | 1.001 | 642.5 |
| 25.75 | 0.861 | 0.936 | 1.038 | 666.6 |
| 26 | 0.854 | 0.923 | 1.022 | 673.2 |
| 26.25 | 0.867 | 0.915 | 1.035 | 691.5 |
| 26.5 | 0.842 | 0.921 | 1.015 | 688.6 |
| 26.75 | 0.848 | 0.929 | 1.027 | 684.5 |
| 27 | 0.851 | 0.924 | 1.018 | 673.2 |
| 27.25 | 0.859 | 0.917 | 1.023 | 691.2 |
| 27.5 | 0.862 | 0.925 | 1.029 | 664.4 |
| 27.75 | 0.867 | 0.923 | 1.032 | 682.5 |
| 28 | 0.854 | 0.919 | 1.044 | 699.6 |
| 28.25 | 0.857 | 0.927 | 1.021 | 681.2 |
| 28.5 | 0.859 | 0.914 | 1.039 | 688.6 |
| 28.75 | 0.864 | 0.905 | 1.064 | 690.2 |
| 29 | 0.861 | 0.901 | 1.051 | 682.5 |
| 29.25 | 0.857 | 0.891 | 1.045 | 679.5 |
| 29.5 | 0.854 | 0.914 | 1.069 | 684.2 |
| 29.75 | 0.861 | 0.918 | 1.075 | 687.2 |
| 30 | 0.855 | 0.914 | 1.065 | 690.8 |

| Fig. 6. Induced Airflow Characteristics and Dust Emission with different bunker height and feeding speed | | | | | | |
| --- | --- | --- | --- | --- | --- | --- |
| Bunker height | Induced airflow velocity | Induced airflow velocity2 | Induced airflow velocity3 | Dust concentration | Dust concentration2 | Dust concentration3 |
| m | m/s | m/s | m/s | mg/m3 | mg/m3 | mg/m3 |
|  | Induced airflow velocity at X3=2t/h | Induced airflow velocity at X3=5t/h | Induced airflow velocity at X3=8t/h | Dust concentration at X3=2t/h | Dust concentration at X3=5t/h | Dust concentration at X3=8t/h |
| 0.75 | 0.59 | 0.65 | 0.69 | 616.4 | 746.8 | 880.8 |
| 0.95 | 0.66 | 0.74 | 0.84 | 714.2 | 860.2 | 928.4 |
| 1.15 | 0.79 | 0.84 | 0.91 | 807.6 | 965.8 | 1091.7 |

Figures 7 and 8 and 9 use the data in Table 2

| Fig. 10. The theoretical curve and measured curve correlation of induced airflow velocity | | | | |
| --- | --- | --- | --- | --- |
| Test number | Experiment | Hemeon model | Li model | 2FI model |
|  | Induced airflow velocity |  |  |  |
|  | m/s | % | % | % |
| 1 | 1.675 | 0.0008 | -0.42177 | -0.06244 |
| 2 | 1.603 | 0.0728 | -0.34977 | 0.00154 |
| 3 | 1.574 | -0.14121 | -0.47013 | -0.14558 |
| 4 | 1.869 | -0.1932 | -0.61577 | -0.11605 |
| 5 | 1.543 | -0.36821 | -0.73526 | -0.13968 |
| 6 | 1.221 | 0.00402 | -0.53108 | 0.03246 |
| 7 | 1.846 | -0.25156 | -0.81153 | -0.12617 |
| 8 | 1.598 | 0.3466 | 1.16169 | 0.00537 |
| 9 | 1.341 | -0.28531 | -0.47906 | -0.08871 |
| 10 | 1.281 | -0.01956 | -0.08832 | -0.01164 |
| 11 | 1.371 | 0.06179 | 0.78384 | 0.11441 |
| 12 | 1.722 | -0.12756 | -0.68753 | -0.13453 |
| 13 | 1.279 | -0.22331 | -0.41706 | -0.03473 |
| 14 | 1.912 | 0.25198 | 0.67417 | -0.01721 |
| 15 | 0.899 | 0.0036 | -0.36029 | 0.03795 |
| 16 | 1.337 | 0.09579 | -0.55373 | 0.1006 |
| 17 | 1.377 | -0.01378 | -0.73046 | 0.03551 |
| 18 | 1.725 | -0.29221 | -0.62113 | -0.1562 |
| 19 | 1.176 | 0.08544 | 0.01668 | -0.039 |
| 20 | 1.993 | -0.12814 | -0.81856 | -0.08902 |
| 21 | 0.907 | 0.17152 | -0.16157 | 0.05504 |
| 22 | 1.649 | -0.05456 | -0.61453 | -0.06956 |
| 23 | 1.198 | 0.39644 | -0.16353 | 0.52985 |
| 24 | 1.334 | 0.09879 | 0.82084 | 0.08523 |
| 25 | 1.121 | 0.10402 | -0.43108 | 9.10E-05 |
| 26 | 1.831 | 0.1136 | 0.92869 | -0.02106 |
| 27 | 0.927 | -0.0244 | -0.38829 | 0.07613 |
| 28 | 1.114 | 0.14744 | 0.07868 | 0.01498 |
| 29 | 1.797 | -0.1212 | -0.54377 | -0.05207 |
| 30 | 1.779 | 0.49541 | 1.35408 | 0.0747 |
| 31 | 1.789 | 0.1556 | 0.97069 | -0.04524 |
| 32 | 1.863 | 0.41141 | 1.27008 | 0.05688 |
| 33 | 1.175 | -0.11931 | -0.31306 | -0.0631 |
| 34 | 1.466 | -0.03321 | -0.36213 | -0.04561 |
| 35 | 1.526 | 0.4186 | 1.23369 | 0.06935 |
| 36 | 1.114 | -0.05831 | -0.25206 | -0.01012 |
| 37 | 1.344 | -0.08256 | -0.15132 | -0.06661 |
| 38 | 1.278 | -0.05298 | -0.58808 | -0.01652 |
| 39 | 1.354 | 0.07879 | -0.25013 | -0.06597 |
| 40 | 1.718 | 0.2266 | 1.04169 | 0.01774 |
| 41 | 1.064 | 0.16102 | -0.37408 | 0.04907 |
| 42 | 0.953 | -0.02356 | -0.02172 | -0.000148 |
| 43 | 1.1176 | 0.24562 | -0.47106 | 0.15452 |
| 44 | 1.409 | 0.06639 | -0.05494 | 0.04451 |
| 45 | 1.337 | 0.37504 | 1.6447 | 0.10732 |
| 46 | 2.266 | -0.83321 | -1.16213 | -0.82956 |

| Fig. 11. Standard deviation of induced airflow velocity box and whiskers | | |
| --- | --- | --- |
| Hemeon model | Li model | 2FI model |
| Standard deviation | Standard deviation | Standard deviation |
| 0.0005677 | 0.29823 | 0.04415 |
| 0.05148 | 0.24732 | 0.00109 |
| 0.09985 | 0.33243 | 0.10294 |
| 0.13661 | 0.43541 | 0.08206 |
| 0.26036 | 0.51991 | 0.09877 |
| 0.00284 | 0.37553 | 0.02295 |
| 0.17788 | 0.57384 | 0.08921 |
| 0.24508 | 0.39717 | 0.0038 |
| 0.20174 | 0.33875 | 0.06273 |
| 0.01383 | 0.06245 | 0.00823 |
| 0.04369 | 0.55426 | 0.0809 |
| 0.0902 | 0.48616 | 0.09513 |
| 0.1579 | 0.29491 | 0.02456 |
| 0.17818 | 0.19387 | 0.01217 |
| 0.00255 | 0.25476 | 0.02683 |
| 0.06773 | 0.39155 | 0.07114 |
| 0.00974 | 0.51651 | 0.02511 |
| 0.20662 | 0.4392 | 0.01104 |
| 0.06042 | 0.0118 | 0.02758 |
| 0.09061 | 0.57881 | 0.06295 |
| 0.12128 | 0.11425 | 0.03892 |
| 0.03858 | 0.43454 | 0.04918 |
| 0.28032 | 0.11563 | 0.03747 |
| 0.06986 | 0.58042 | 0.06026 |
| 0.07355 | 0.30482 | 6.43E-05 |
| 0.08033 | 0.23242 | 0.01489 |
| 0.01725 | 0.27456 | 0.05383 |
| 0.10426 | 0.05564 | 0.01059 |
| 0.0857 | 0.3845 | 0.03682 |
| 0.35031 | 0.25283 | 0.05282 |
| 0.11002 | 0.1914 | 0.03199 |
| 0.29091 | 0.17355 | 0.04022 |
| 0.08437 | 0.22137 | 0.04462 |
| 0.02348 | 0.25606 | 0.03225 |
| 0.29599 | 0.51879 | 0.04904 |
| 0.04123 | 0.17823 | 0.00716 |
| 0.05838 | 0.107 | 0.0471 |
| 0.03746 | 0.41583 | 0.01168 |
| 0.05571 | 0.17687 | 0.04665 |
| 0.16023 | 0.31232 | 0.01254 |
| 0.11386 | 0.26451 | 0.0347 |
| 0.01666 | 0.01536 | 1.05E-04 |
| 0.17368 | 0.33309 | 0.09262 |
| 0.04694 | 0.03885 | 0.03147 |
| 0.26519 | 0.58917 | 0.07589 |
| 0.16208 | 0.39465 | 0.0595 |

Figure 12 uses the data in Table 5
